# Virtual Reality as a Therapy Adjunct for Fear of Movement in Veterans With Chronic Pain: Single-Arm Feasibility Study

**DOI:** 10.2196/11266

**Published:** 2019-10-30

**Authors:** Christopher A Fowler, Lisa M Ballistrea, Kerry E Mazzone, Aaron M Martin, Howard Kaplan, Kevin E Kip, Katherine Ralston, Jennifer L Murphy, Sandra L Winkler

**Affiliations:** 1 Research and Development Service James A Haley Veterans Hospital Tampa, FL United States; 2 Chronic Pain Rehabilitation Program James A Haley Veterans Hospital Tampa, FL United States; 3 Advanced Visualization Center Information Technology University of South Florida Tampa, FL United States; 4 College of Public Health University of South Florida Tampa, FL United States; 5 Department of Neurology College of Medicine University of South Florida Tampa, FL United States

**Keywords:** chronic pain, virtual reality, Veterans, pain management, rehabilitation, fear of movement, kinesiophobia, exposure therapy, distraction therapy

## Abstract

**Background:**

Virtual reality (VR) has demonstrated efficacy for distraction from pain-related thoughts and exposure to feared movements. Little empirical VR research has focused on chronic pain management.

**Objective:**

The purpose of this study was to examine the feasibility of VR as an adjunctive intervention for Veterans with chronic pain. We designed a hierarchy ranging from low-intensity pain distraction to high-intensity movement-based exposure for this purpose. VR apps were mapped onto the hierarchy.

**Methods:**

Sixteen Veterans receiving inpatient chronic pain rehabilitation participated in daily VR sessions over a 3-week period. Trajectories across the distraction-to-exposure hierarchy and Veteran-reported intensity ratings were described and evaluated over time. Minimum clinically important differences (MCIDs), pre-post effect sizes, and 95% confidence intervals were examined for fear of movement using the Fear of Daily Activities Questionnaire (FDAQ) and Pain Outcomes Questionnaire-VA (POQ-VA; fear scale). This approach was applied to secondary outcomes: POQ-VA (pain intensity, interference, negative affect), Pain Catastrophizing Scale, and Patient-Specific Functioning Scale (PSFS). Session attendance, completion, and VR experiences were described.

**Results:**

Ten of 14 Veterans (71%) who participated in three or more VR sessions completed the distraction-to-exposure hierarchy. Only three trajectories emerged more than once. Due to high completion rates, Veterans that completed the hierarchy could self-select nonhierarchy apps. Veterans rated all hierarchy levels (low, medium, high) near medium intensity. Self-selected activities were rated as high intensity. For kinesiophobia, six Veterans (38%) exceeded the MCID on the FDAQ and a small effect size improvement was observed (Cohen d=−0.35). The confidence interval (95% CI −0.71 to 0.01) indicated the possibility of a null effect. The POQ-VA fear scale yielded no effect (Cohen d=0.06, 95% CI −0.43 to 0.54). For secondary outcomes, Veterans exceeding MCID were calculated with complete data: pain intensity (1/15, 7%), pain catastrophizing (5/14, 36%), and patient-specific functioning (10/15, 67%). Effect sizes were large for patient-specific functioning (Cohen d=1.14, 95% CI 0.50-1.78), medium for mobility interference (Cohen d=−0.56, 95% CI −0.96 to −0.16), and small for pain intensity (Cohen d=−0.40, 95% CI −0.69 to −0.12) and catastrophizing (Cohen d=−0.41, 95% CI −0.79 to −0.02). No effects were observed for interference in daily activities (Cohen d=0.10, 95% CI −0.27 to 0.47) and negative affect (Cohen d=0.07, 95% CI −0.26 to 0.40). Veterans attended 85.2% (98/108) of VR sessions and completed 95% (93/96) of sessions attended. Twenty-minute sessions were rated as too short. No significant adverse events were reported.

**Conclusions:**

Findings support the feasibility of VR as an adjunct for Veterans with chronic pain. However, the hierarchy will require modification, as evidenced by homogeneous intensity ratings. Veteran-selected activities presented the highest intensity ratings, largest outcome effect size (PSFS), and MCID. This highlights the important role of utilizing Veteran stakeholders in hierarchy modification, design of VR interventions, and outcome selection.

## Introduction

### Virtual Immersion

Virtual environments present an opportunity to safely and gradually expose Veterans with chronic pain to movements they avoid in the real world. Virtual reality (VR) describes “computer-generated simulations of three-dimensional objects or environments with seemingly real, direct, or physical user interaction” [1]. VR technologies use wearable devices to project a virtual environment and to track movements within it [2]. Sensory inputs (eg, visual, audio, tactile) give the user the illusion of “immersion” or being cognitively absorbed by a virtual environment [3,4]. Interaction with the immersive environment allows the user to co-create their experience giving them a subjective sense of “presence” in the virtual environment [3,5]. Immersion and presence can facilitate interventions using simulated 3D environments that set VR apps apart from in vivo behavioral treatments [6] and low-immersion 2D apps (eg, mobile phone) [7]. VR can serve as an adjunct to assist with the adoption of pain management skills in evidence-based interventions [4,8].

### Virtual Reality for Pain Management

Virtual reality apps have demonstrated efficacy and feasibility for delivering pain management skills, including distraction and exposure therapies. A rapid review (20 studies, N=337) found short-term pain reduction (strong evidence) and analgesic effects (moderate evidence) [4]. A meta-analysis of controlled studies (16 studies, N=656) estimated a medium effect size pain reduction when using VR during medical procedures [9]. To date, VR research has prominently focused on acute, not chronic, pain [4,9].

### Chronic Versus Acute Pain

Pain is defined as “an unpleasant sensory and emotional experience associated with actual or potential tissue damage or described in terms of such damage” [10]. Postinjury acute pain is a nociceptive physiological warning to limit or avoid certain behaviors to prevent further harm [11]. Acute pain lasts 3 months or less and helps facilitate physical healing [12]. In chronic pain, generalized hypersensitivity in the central nervous system results in overactive pain [13] and sensory [14] pathways. Chronic pain persists beyond the physical healing process (ie, >3 months) and serves no adaptive purpose [12]. Still, chronic pain may feel indistinguishable from acute pain and leads people to avoid movement, which negatively impacts functioning [13,15].

### Fear of Movement

The fear-avoidance model of chronic pain posits a feedback loop with fear as a key component of avoidant behavior [16]. People with chronic pain mistakenly believe that pain sensations signal harm. Cognitive biases in pain processing (eg, behaviors that aggravate pain should be avoided to prevent reinjury) can result in kinesiophobia, or fear of movement, and subsequent pain avoidance as a means of self-protection [16,17]. Avoidance promotes a self-perpetuating cycle of physical deconditioning, negative affect, disability, and worse pain [17-19]. Interventions that operate on fear-avoidance principles (eg, graded physical therapy, cognitive behavioral therapy) aim to disrupt this cycle through gradual exposure to feared movements [15,20]. Reengagement in feared movements can modify pain interpretations (eg, pain signals harm), disrupt fear avoidance, and combat physical deconditioning [20-22]. This is often challenging because even safe movement can cause pain and emotional discomfort.

### Pain Distraction

The most common VR intervention has been distraction therapy [4,9] not exposure therapy. Distraction is hypothesized to be a mechanism of action for VR in attenuating pain [23]. Distraction therapy is based on the assumption that people have finite cognitive resources for information processing [24]. Immersive VR consumes cognitive and attentional resources through sensory input, thereby limiting pain-processing capabilities [23,25]. A meta-analysis (14 studies, N=581) found large effect size reductions in acute and laboratory-induced pain when using VR distraction versus controls [26].

Distraction is important in the cognitive behavioral therapy for chronic pain protocol of Veterans Affairs (VA) [15]. A paucity of studies supports VR distraction for chronic pain. A within-group pilot study found a large effect size improvement in pain intensity following VR use [27]. A randomized crossover study found reduced pain intensity during VR compared with self-mediated distraction (eg, meditation, gaming), but not at posttest [28]. Still, VR provided superior distraction than self-mediated methods as evidenced by 56% less time thinking about pain [28]. Thus, VR distraction may be beneficial for chronic pain management.

### Graded Exposure

People with chronic pain benefit from rehabilitation, not just immediate relief [8,22]. As such, passive pain distraction and more activating therapies, including exposure to feared movements, are used in comprehensive protocols [15]. Exposure therapy is important for chronic pain rehabilitation and compatible with VR [8,22]. A randomized controlled trial (RCT) assigned people with acute and chronic low back pain to receive 10 sessions of physical therapy with or without VR exposure (“virtual walking”) [29]. Participants who received VR before each session experienced medium-to-large improvements in kinesiophobia, walking distance, and disability. A feasibility study of VR dodgeball found increased spine flexion, no adverse events, high acceptability, and likelihood to recommend the game to others with chronic back pain [30]. One study found that 76% of participants indicated a preference for VR exposure therapy over in vivo exposure before randomization [31]. Only 3% indicated that they would refuse VR compared with 27% for in vivo. This evidence supports VR exposure as an efficacious and acceptable adjunct for chronic pain management.

### Distraction-to-Exposure Hierarchy

This study was informed by the fear-avoidance model of chronic pain [16], assuming that gradual exposure to feared movements using VR can improve kinesiophobia and pain outcomes. Still, people with chronic pain experience sensory exacerbation which may be a VR contraindication. Passive distraction therapy apps may be useful to gradually integrate Veterans to VR use. Thus, we created a hierarchy of increasing intensity based on sensory integration theory [32]. Veterans began with low movement-intensive VR distraction apps and could gradually progress to medium and high movement-intensity apps (exposure). This helps ensure that VR does not over- or understimulate the user.

### Gaps Addressed

This study addresses notable gaps in VR research. First, there is a dearth of chronic pain studies in the growing literature about VR for pain management [9,33]. Second, VR studies have examined individual applications of pain distraction and exposure. Despite the efficacy of both therapies and distraction inherent in VR, no identified studies used a hierarchy approach [23]. Third, we failed to identify any VR pain management studies conducted with Veterans. More research is needed given the need for alternative nonpharmacological treatments for Veterans [34], who experience chronic pain with greater prevalence than non-Veterans [35].

### Specific Aims

This study examined the feasibility of VR as an adjunct for chronic pain management. Evidence obtained is intended to inform a future RCT that will test the efficacy of VR and aim to validate our current and future revised distraction-to-exposure hierarchy. Primary aims are to (1) describe and compare the Veteran trajectories and self-reported app intensity ratings over a 3-week treatment period on the distraction-to-exposure hierarchy, (2) estimate the proportion of Veterans experiencing minimum clinically important differences (MCIDs) and within-subject effect size and 95% confidence intervals (CIs) for fear of movement and secondary pain outcomes associated with the use of VR, and (3) pilot test this protocol to assess the feasibility of VR use to plan for a future RCT.

## Methods

### Study Setting

This study was conducted in the Chronic Pain Rehabilitation Program at the James A Haley Veterans Hospital in Tampa, FL. This program used a cognitive behavioral approach to target the biopsychosocial impact of chronic pain [36]. Veterans were referred to this unique 19-day residential treatment program from throughout the VA system. For a more comprehensive overview of this program, see Murphy and colleagues [36,37].

### Participants and Recruitment

Each week, up to four Veterans entered the 19-day Chronic Pain Rehabilitation Program, and up to four graduated and were discharged. All Veterans (N=18) that entered the program over the 3-week study period were targeted for recruitment; one Veteran refused. Of the 17 Veterans that consented, one did not meet the inclusion criteria. The final sample consisted of 16 Veterans (89%). Inclusion criteria were (1) diagnosis of chronic pain syndrome (*International Classification of Diseases [ICD], Ninth Revision, Clinical Modification* code 338.4 and *ICD-10* code G89.4) and (2) negative screen for illicit substances and unprescribed opioids. Exclusion criteria were uncontrolled medical and psychological factors (ie, aggression, depression, psychosis, suicidality) that could interfere with rehabilitation. These criteria mirrored that of the chronic pain program [36,37].

### Design

A hybrid type 1 implementation-effectiveness design [38] was used to collect clinical efficacy and preimplementation data for VR. Qualitative and quantitative preimplementation data were collected following each VR session (aims 1 and 3). This paper emphasizes quantitative outcomes. Fear of movement and pain outcomes were assessed using a within-participants pretest-posttest design (aim 2).

### Intervention

**Figure 1:**
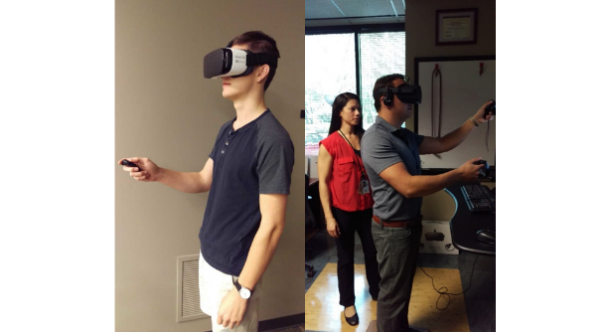
Samsung Oculus Gear VR with supplemental hand controller (left) and Oculus Rift (right).

The distraction-to-exposure hierarchy was built with input from chronic pain program clinical stakeholders [39]. The hierarchy started with low stimulation intensity and then moved to high movement intensity. Twelve commercially available VR apps, six per head-mounted display (HMD), were then chosen to fit intensity levels. Low-intensity distraction apps included mindfulness meditation [40] and visual imagery [41,42], which required minimal movement. Medium-intensity apps included virtual walking or swimming [43,44] and controlling aircraft or watercraft [45,46], which required head and neck movement. High-intensity apps were 3D painting [47,48] and music or rhythmic-based [49,50], which also required torso and upper extremity movement. Veterans alternated between two commercially available VR HMDs: Oculus Rift [51] and Samsung Oculus Gear VR [52]. Rift is an HMD with hand-tracking controllers, which is used with commercial gaming computers. Gear VR uses Samsung Galaxy Series mobile phones (S6 and above) to project virtual environments with sound. Both HMDs have been used for pain management research in medical settings [28,53,54]. Figure 1 shows our team members using each HMD.

### Primary Measures

#### Daily Rating Form

The eight-item daily rating form was created by the investigators to assess VR feasibility. Following each VR session, this form was used to track Veteran progress across the hierarchy (app selection), which HMD they used, and the number of sessions attempted and completed. Veterans reported their level of immersion using a single ordinal item adapted from Cole et al [55]: 1=aware and acknowledge the role of technology; 2=partially aware of technology, but perceived being inside a virtual environment; and 3=unaware of technology (complete virtual immersion). In addition, self-reported VR intensity (1=low, 2=medium, 3=high) and VR session length (1=too short, 2=just right, 3=too long) followed a similar approach [55]. Veterans could also provide feedback about their VR experience via three open-ended questions: likes, dislikes, and additional comments. This helped identify any facilitators (eg, HMD preferences), barriers (physical, psychological discomfort), and adverse events (eg, cybersickness, falls).

#### Fear of Movement

Kinesiophobia was assessed using two measures: the Pain Outcomes Questionnaire-VA (POQ-VA) [56] and Fear of Daily Activities Questionnaire (FDAQ) [57]. These scales had modest convergent validity (*r*=.29, *P*=.28) suggesting they could be examined separately. Both scales have demonstrated acceptable psychometric properties in chronic pain studies [56,57].

The POQ-VA [56] is a multidimensional instrument developed specifically for the Veteran population. The fear subscale measures kinesiophobia using two items (fear of reinjury, safe to exercise) on Likert-type scales ranging from 0 to 10 and summed, with higher scores indicating positive outcomes. No MCID standards were identified.

The 10-item FDAQ [57] was designed in accordance with the fear-avoidance model. It was used to assess common feared movements (eg, sitting, standing, lifting, walking). All items are measured on scales anchored by 0 (no fear) and 100 (maximal fear) and then averaged. The MCID for the FDAQ is a 12.90-point reduction from baseline [57].

### Secondary Measures

#### Pain Outcomes Questionnaire-VA

Secondary outcomes were examined to identify promising outcomes for use with a future VR RCT [39]. Multiple secondary pain outcomes were collected using the POQ-VA [56]. These were interference with activities of daily living and mobility as well as negative affect. No MCID scores were identified. The POQ-VA also assesses pain intensity using the common pain Numeric Rating Scale [58] (0=no pain at all to 10=worst possible pain). The Numeric Rating Scale MCID is 2.10 points for moderate pain (baseline=4-6) and 2.80 points for severe pain (baseline ≥7) [59].

#### Pain Catastrophizing Scale

The 13-item Pain Catastrophizing Scale [60] was used to measure exaggerated beliefs about pain (eg, nothing I can do to reduce pain). Items are measured on a Likert-type scale anchored by 0 (not at all) and 4 (very) and summed with higher scores indicating maladaptive beliefs about pain. MCIDs of 38% or greater have been established for improved disability and pain intensity following pain rehabilitation [61].

#### Patient-Specific Functional Scale

The Patient-Specific Functional Scale (PSFS) [62] required Veterans to identify three activities that have been hindered because of their pain. These tasks were then rated 0 (unable to perform) to 10 (able to perform at prior level) and averaged. The MCID for the PSFS is 1.30 to 2.29 points (small), 2.30 to 2.69 (medium), and 2.70 or higher (large) [63].

### Procedures

Veterans were informed about the study during orientation to the pain program. Consenting procedures were performed in-person by the research team before their first physical therapy session. All study procedures were approved by the James A Haley VA Research and Development Committee and the University of South Florida (Tampa) Institutional Review Board (protocol: 00031503).

Veterans completed 20 minutes of VR during daily physical therapy sessions. During session 1, the hierarchy was described to the Veterans, and they began with low intensity guided meditation. Each session, they were asked which intensity VR APPS they would like to use that session. Following each session, research staff administered the daily rating form. Primary and secondary outcome measures were administered to Veterans at intake and discharge (approximately 3 weeks) to the chronic pain program to track improvements. The research staff retrieved these data from the VA’s electronic medical record.

### Statistical Analysis

To address aim 1 (describe and compare the Veteran trajectories and self-reported app intensity ratings on the distraction-to-exposure hierarchy), distributions of the Veteran-selected apps (proposed intensity range 1-3) were plotted across VR sessions. The frequency of Veteran trajectories toward completing the hierarchy were counted to identify common patterns. Veterans that completed less than three VR sessions were excluded from this analysis because the hierarchy could not be completed in two sessions. Veterans’ median self-reported app intensity ratings were also calculated and plotted across the first nine VR sessions. Sessions 10 and 11 were excluded due to the low frequency of Veterans (N≤2) that attended more than nine sessions. Consistencies across proposed and self-reported VR app intensity were descriptively compared.

For aim 2 (estimate the proportion of Veterans experiencing MCIDs and within-subject effect size and 95% CI for fear of movement and secondary pain outcomes associated with the use of VR), changes in fear of movement and secondary outcomes were calculated and compared with established MCID scores for each respective measure excluding imputed missing values. The proportion of Veterans that exceeded MCID was calculated for each outcome. Within-participants Cohen *d* effect sizes ([post mean−pre mean]/SD difference) and 95% CI for fear of movement and secondary outcomes were calculated to examine the efficacy of VR [64,65]. Suspected outliers were assessed using multiple criteria. These included examining boxplots of these pre-to-post test change scores, Tukey fences [66], and clinical observation and consultation noted during the study. In the case of a suspected outlier, findings were reported with and without its inclusion.

For aim 3 (pilot test this protocol to assess the feasibility of VR use), compliance for the Veteran sample was calculated via the proportion of VR sessions attended (sessions attended/total scheduled sessions). Adherence was calculated using the proportion of completed 20-minute VR sessions (full sessions completed/sessions attended). Veteran ratings of the length of the session and their self-reported levels of immersion were plotted across sessions.

### Availability of Data and Materials

The final deidentified datasets from this study (qualitative and quantitative) and the VR user manual will be made available by the corresponding author on reasonable request.

## Results

### Demographic Characteristics

Veterans ranged from 28 to 63 years, with a mean age of 49 (SD 12) years (Table 1). They were prominently male, and their racial/ethnic composition was primarily Caucasian or white. Median pain duration was 16.50 (IQR 14.62) years and baseline pain intensity was near the severe range (≥7) on the Numeric Rating Scale [38]. The primary pain location was low back; daily opioid use was low.

**Table 1:** Demographic characteristics for study sample (N=16).

Characteristic		Participants
Age (years), mean (SD)		48.88 (11.62)
**Gender,** **n ** **(%)**		
	Female	3 (19)
	Male	13 (81)
**Ethnicity, n (%)**		
	African American or black	4 (25)
	Caucasian or white	8 (50)
	Hispanic or Latino	2 (12)
	Other	2 (13)
**Pain location, n (%)**		
	Head	2 (13)
	Low back	11 (69)
	Other	3 (19)
Pain chronicity (years), median (IQR)		16.50 (14.62)
Virtual reality sessions, median (IQR)		7.50 (6.50)

### Aim 1: Describe and Compare the Veteran Trajectories and Self-Reported App Intensity Ratings on the Distraction-to-Exposure Hierarchy

**Figure 2:**
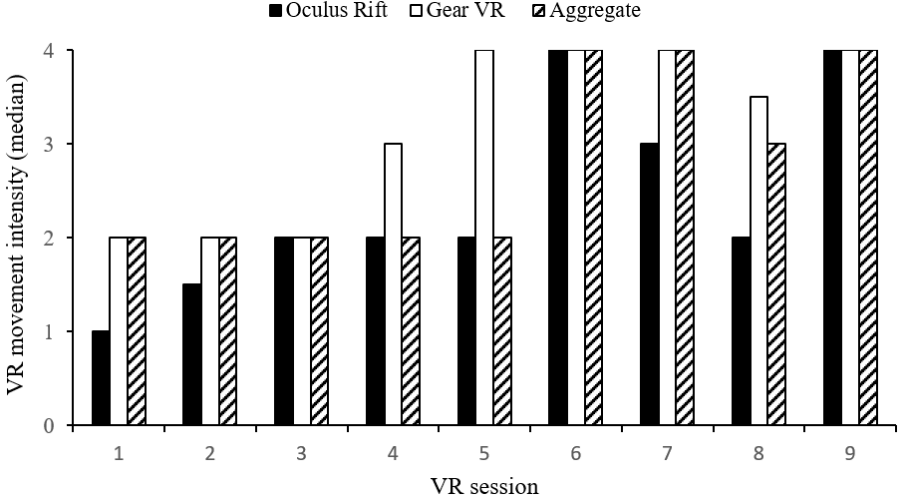
Median Veteran progression across the distraction-to-exposure hierarchy. App movement intensity: 1=low, 2=medium, 3=high, 4=self-selected. VR: virtual reality.

In total, 10 of 14 Veterans (71%) who participated in three or more VR sessions completed the hierarchy. Eleven different trajectories emerged among these 14 Veterans during completion of the hierarchy. Only three patterns (21%) occurred more than once, which indicated notable variability. Six of the initial eight Veterans (75%) reached the highest level of the hierarchy during the first week of testing. Because of this high frequency early in the study, Veterans that completed the hierarchy could self-select VR activities (eg, fishing, basketball free throws) in addition to hierarchy apps. Self-selected apps were assigned a fourth intensity level indicating they were beyond the hierarchy. Median progressions across the distraction-to-exposure hierarchy are presented in Figure 2.

**Figure 3:**
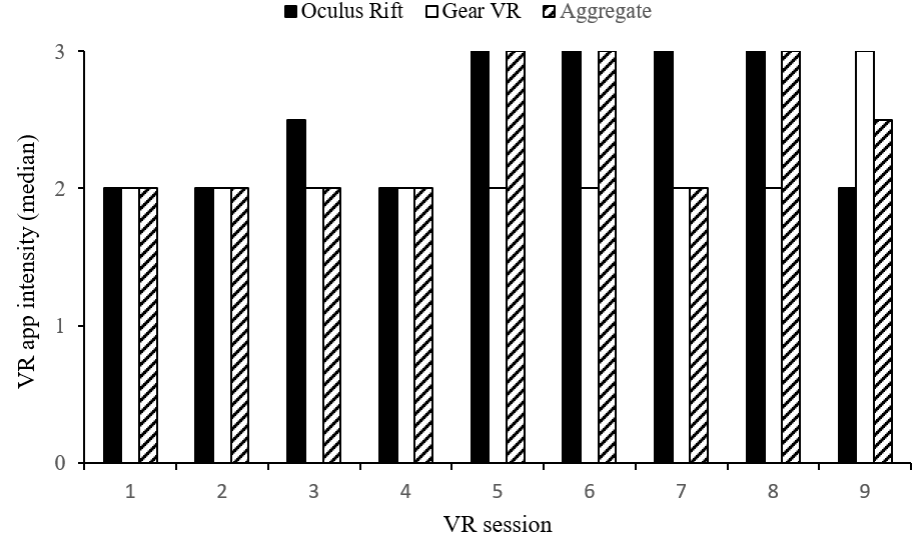
Veteran-reported intensities for virtual reality (VR) apps across sessions. Self-reported app intensity: 1=low, 2=medium, 3=high.

**Figure 4:**
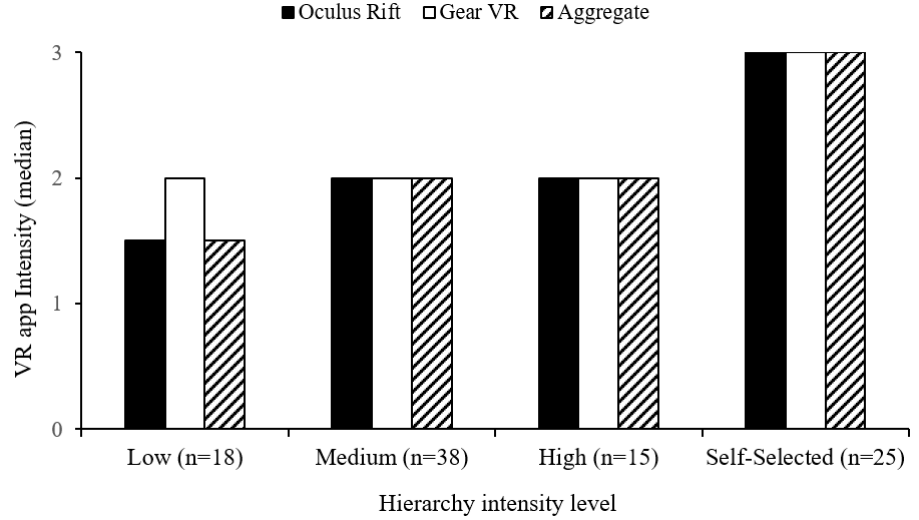
Veteran-reported intensities for virtual reality (VR) apps across movement intensity levels.

Veterans rated the intensity of each VR session. Low- to medium-intensity ratings were reported for each of the first four sessions (median 2, IQR 1.5). Sessions five to nine were rated from medium to high intensity (median 3, IQR 1; see Figure 3). Comparisons between Veterans’ self-reported intensity ratings and hierarchy movement intensity levels were examined. Veterans rated low movement intensity apps (ie, distraction) between low and medium intensity (median 1.5, IQR 1). Both medium- (median 2, IQR 1) and high-intensity (median 2, IQR 1) movement exposure apps were rated as medium intensity. Veteran-selected apps were rated as high intensity (median 3, IQR 1). Veteran intensity ratings across hierarchy levels are presented in Figure 4.

### Aim 2: Estimate the Effect Size, 95% CI, and Proportion of Veterans Experiencing Minimum Clinically Important Difference for Fear of Movement and Pain Outcomes With Virtual Reality Use

For the primary outcome kinesiophobia, 10 of 16 Veterans (63%) had improved scores from baseline with six (38%) exceeding an MCID of 12.9 points or greater [58]. The observed effect size improvement was minimal (ie, Cohen *d*<0.20) on the FDAQ (Cohen *d*=−0.15, 95% CI −0.55 to 0.25). One Veteran was identified as a possible outlier. When this score was excluded, a small effect size reduction in fear of movement was observed (Cohen *d*=−0.35, 95% CI −0.71 to 0.01). However, there was notable variability in the confidence interval with the effect size ranging from no effect to a medium improvement suggesting a possible null finding.

Using the POQ-VA fear scale, there was little evidence of a reduction in kinesiophobia (Cohen *d*=−0.10, 95% CI −0.69 to 0.48). When a possible outlier score was excluded, the effect size changed directions but did not amount to a notable effect (Cohen *d*=0.06, 95% CI −0.43 to 0.54). Full sample scale scores for primary and secondary measures, effect sizes, 95% CIs, and MCID are presented in Table 2.

**Table 2:** Baseline and posttest scores for fear of movement and secondary outcomes (N=16).

Measure		Baseline	Posttest	Effect size (95% CI)	MCID^a^, n (%)
**Kinesiophobia, mean (SD)**					
	FDAQ^b^	59.20 (24.83)	56.45 (21.02)	−0.15 (−0.55, 0.25)	6 (38)
	POQ-VA^c^ Fear	12.44 (5.01)	11.91 (3.01)	−0.10 (−0.69, 0.48)	N/A^d^
**Outliers excluded^e^**					
	FDAQ	60.28 (25.31)	54.75 (20.58)	−0.35 (−0.71, 0.01)	—^f^
	POQ-VA Fear	12.31 (2.65)	12.07 (4.95)	0.06 (−0.47, 0.59)	—
**Pain outcomes (POQ-VA), mean (SD)**					
	Interfere daily living	16.44 (12.36)	17.33 (12.03)	0.10 (−0.27, 0.47)	N/A
	Interfere mobility	26.31 (9.58)	22.25 (10.82)	−0.56 (−0.96, −0.16)	N/A
	Negative affect	28.56 (10.96)	29.09 (11.87)	0.07 (−0.26, 0.40)	N/A
**Numeric Rating Scale, mean (SD)**					
	Pain intensity	6.88 (1.26)	6.38 (1.59)	−0.48 (−0.87, −0.10)	1 (7)
	Outlier excluded	6.73 (1.16)	6.40 (1.64)	−0.40 (−0.69, −0.12)	—
PCS^g^		28.83 (10.39)	24.54 (15.45)	−0.41 (−0.79, −0.02)	5 (36)
PSFS^h^		3.60 (1.59)	5.98 (2.37)	1.14 (0.50, 1.78)	10 (67)

^a^MCID: minimum clinically important difference.

^b^FDAQ: Fear of Daily Activities Questionnaire.

^c^POQ-VA: Pain Outcomes Questionnaire-VA.

^d^Not applicable.

^e^N=15.

^f^MCID not recalculated.

^g^PCS: Pain Catastrophizing Scale.

^h^PSFS: Patient-Specific Functioning Scale.

### Aim 2: Secondary Outcomes

The POQ-VA pain interference with mobility and activities of daily living as well as negative affect scales were examined. Veterans experienced a medium effect size improvement in interference with mobility (Cohen *d*=−0.56, 95% CI −0.96 to −0.16). There was variability with the effect size interval ranging from a small to large effect that was statistically significant (*P*<.05) because the interval did not contain the null value of zero. Conversely, Veterans experienced a slight exacerbation in interference with activities of daily living (Cohen *d*=0.10, 95% CI −0.27 to 0.47). A similar pattern was observed with negative affect (Cohen *d*=0.07, 95% CI −0.26 to 0.40). Intervals for the latter two findings suggested no effect.

Five of 15 Veterans (33%) with complete data had reduced pain intensity scores from baseline using the Numeric Rating Scale. However, only one Veteran (7%) met MCID of 2.10 points or greater for moderate pain intensity at baseline (rating=4-6) [59]. No Veterans with severe baseline pain (rating ≥7) exceeded the MCID [59]. The sample had a small to medium improvement in pain intensity (Cohen *d*=−0.49, 95% CI −0.87 to −0.11). The confidence interval suggested a minimal-to-large effect. When a possible outlier was excluded, a similar effect pattern remained (Cohen *d*=−0.40, 95% CI −0.69 to −0.12). These effects were statistically significant (*P*<.05) because the intervals did not contain the null value of zero.

Ten of 14 Veterans (71%) had reduced catastrophizing scores from baseline, and five (36%) exceeded the MCID of 38% or greater improvement [61]. Veterans experienced a small to medium effect size improvement in pain catastrophizing (Cohen *d*=−0.41, 95% CI −0.79 to −0.02). The interval ranged from a minimal to large and was statistically significant.

Finally, 14 of 15 Veterans (93%) reported improvements in patient-specific functional tasks that were previously hindered by their pain, as measured by the PSFS. Ten Veterans (67%) exceeded the MCID. Based on the scheme described by Abbott and Schmitt [63], Veterans MCID improvements were categorized as small (1.30-2.29; *n*=2), medium (2.30-2.69; *n*=2), and large (≥2.70; *n*=6). The observed effect size improvement in patient-specific functioning ranged from medium to large and was statistically significant (Cohen *d*=1.14, 95% CI 0.50-1.78, *P*<.001).

### Aim 3: Pilot Test the Protocol to Assess the Feasibility of VR Use

Quantitative feasibility outcomes included levels of VR compliance, adherence, and session experiences. The compliance rate for this study (85.2%) was calculated via the number of VR sessions Veterans attended (n=98) divided by the total number of scheduled sessions (n=115). Of the 98 sessions attended, Veterans completed the full 20 minutes in 93 for an estimated adherence rate of 94.9%. The most common reason for missing (n=7) or shortened VR sessions (n=2) was physical therapy-related (eg, longer than expected sessions). Compliance (98/108, 90.7%) and adherence (93/96, 96.9%) rates were calculated accounting for physical therapy as the primary reason for missing VR sessions.

**Figure 5:**
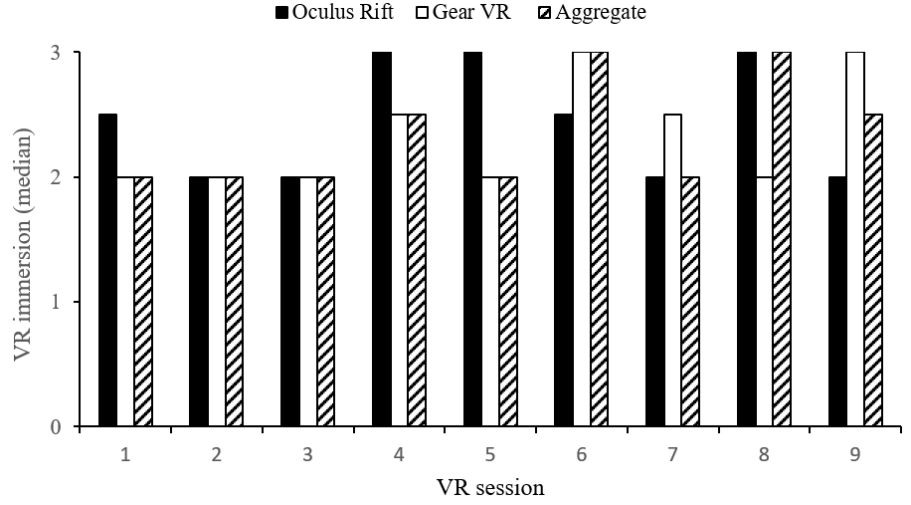
Veteran-rated immersion across virtual reality (VR) sessions. Immersion rating scale: 1=using technology, 2=using technology and immersed in the virtual world, 3= completely immersed in the virtual world.

**Figure 6:**
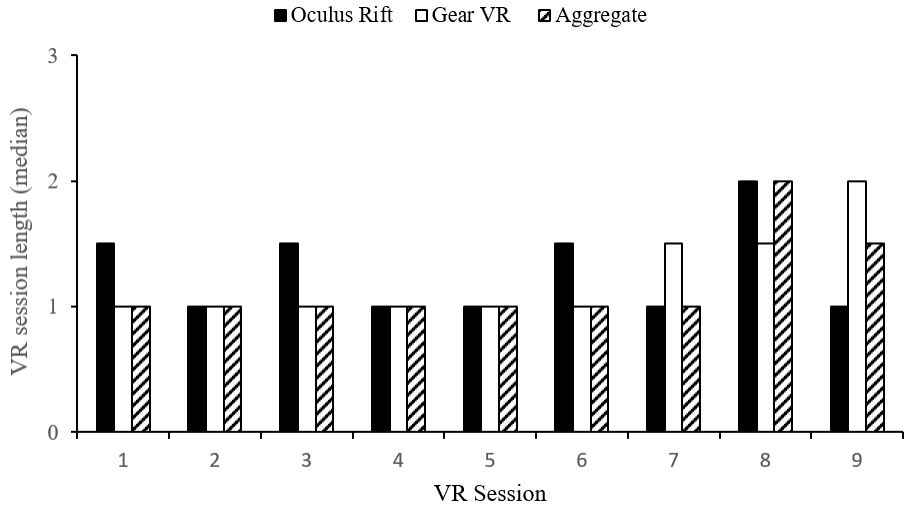
Veteran-rated virtual reality (VR) session length. Session length: 1=too short, 2=just right, 3=too long.

The VR experiences included Veteran-rated immersion, session length, and HMD preferences, and were plotted across sessions. Adverse events were also described. During sessions 1 to 3, the Veteran’s median immersion ratings (median 2, IRQ 1) indicated that they were aware of using technology and were immersed in the virtual world. Sessions 4 to 9 saw ratings vary to include greater immersion in the virtual world with less technology awareness (range 2-3). Overall, median immersion ratings for the Oculus Rift and VR Gear HMDs were equal (median 2, IQR 1). Veterans typically rated Oculus Rift higher across the first five sessions and Gear VR higher across sessions 6 to 9 (Figure 5). Median ratings indicated that 20-minute sessions typically were “too short” (median 1, IQR 1) with no sessions rated lower than “just right.” Overall ratings for each HMD were equal (median 1, IQR 1), although median ratings for Gear VR were occasionally lower (ie, too short) than Oculus Rift across sessions (Figure 6).

No significant adverse events, such as falls, occurred. Minor adverse events included cybersickness symptoms (dizziness: n=2 sessions; nausea: n=4 sessions) and one session being terminated early because the Veteran was “starting to feel the weight [Gear VR] on their neck.” Interestingly, 2 of 16 Veterans (13%) purchased VR headsets during the study. Four additional Veterans (25%) requested VR purchasing information and were provided with a handout (eg, models, cost, reviews) on request.

## Discussion

### Principal Findings

This study examined the feasibility of using a VR distraction-to-exposure hierarchy to improve fear of movement for Veterans with chronic pain. Descriptive findings suggest notable variability in Veteran trajectories across the hierarchy and that exposure apps requiring increased levels of movement were less challenging than anticipated. For the primary outcome (fear of movement), fear of daily activities, which measures specific movements, may be a more promising measure than the POQ-VA fear scale, which contains more general items (ie, exercise, reinjury). Promising pain outcomes for future research include interference with mobility, pain intensity, pain catastrophizing, and patient-specific functional activities. Veterans rated feeling immersed in VR even when they were cognizant of their technology use. They also rated their sessions as too short. Minimal adverse events were reported. This provided favorable evidence for the feasibility of VR for chronic pain management in a VA medical center. However, app selection, particularly for high movement intensity, will require modification before future research to align with the initial hierarchy.

### Trajectories and App Intensity

The first aim of this study was to describe trajectories and self-reported app intensity ratings across the distraction-to-exposure hierarchy. Clinical consultation suggested that the majority of Veterans may not complete the hierarchy due to fear of movement. However, most Veterans reached or surpassed the highest movement level in six sessions. Differences between the Oculus Rift and Gear VR HMDs revealed most Veterans completed the hierarchy after only four sessions using Gear VR versus six sessions using the Oculus Rift. To help ensure Veterans did not lose interest in the study if they found the tasks to be too easy, the hierarchy was expanded to include Veteran-selected activities (eg, fishing, basketball free throws) once they completed the hierarchy.

To support our hierarchy, we anticipated that median self-reported app intensities would align with movement intensity on the same rating scale (1=low, 2=medium, 3=high). Self-reported app intensity ratings suggest that hierarchy apps did not calibrate as expected. Low movement distraction apps were rated more intensely than expected, particularly for Gear VR. Medium movement-intensity apps were rated as expected. Proposed high movement-intensity apps were rated similarly to medium intensity, which indicated that this was not a unique intensity level as anticipated. Similar Veteran ratings across proposed levels of the hierarchy indicate a possible range restriction in movement intensity. Median high intensity scores were not observed until Veterans engaged in self-selected activities. Despite high intensity ratings, post hoc integration of these apps into the distraction-to-exposure hierarchy was difficult due to their heterogeneity.

### Fear of Movement and Pain Outcomes

The second aim was to estimate the effect size, 95% CI, and proportion of Veterans experiencing MCID for fear of movement and pain outcomes. These analyses were to inform design and sample size requirements for a future trial and selection of appropriate instruments for use with VR for chronic pain. The MCID for kinesiophobia indicated that 38% exceeded MCID on the FDAQ. After removal of an outlier, a small effect size improvement was observed for the primary outcome fear of movement using the FDAQ, but not the POQ-VA. However, there was a wide confidence interval associated with these effect sizes (and pain outcomes), which is common in small sample sizes [67].

For secondary pain measures, the MCID statistics, effect size, and confidence interval estimates were generally promising. For pain outcomes, 36% of Veterans exceeded MCID in catastrophizing, but only one met MCID for pain intensity. In total, 67% of Veterans experienced MCID for patient-specific functional outcomes, thus indicating the most promising outcome measure in this small study.

On the POQ-VA, improvements were observed in interference with mobility (medium) and pain intensity (small). The 95% CIs were statistically significant for these outcomes. No changes were observed for negative affect or interference with activities of daily living. There was a small effect size improvement for pain catastrophizing and the significant CI ranged from minimal to large. Similar to MCID, the most promising improvements were for patient-specific functional outcomes. A large effect size was observed in patient-specific functional outcomes with the confidence interval ranging from medium to large.

### Feasibility of Virtual Reality

The final aim was to establish the feasibility of VR as a therapy adjunct. All Veterans completed the study. Session attendance was over 85% and increased to more than 90% after accounting for sessions missed for physical therapy. Adherence, as measured by session completion, was nearly 95% and approximately 97% when accounting for therapy. This adjustment was made because VR was proposed to complement evidence-based interventions, and we did not want to study participation to reduce therapy adherence. Immersion ratings indicated that Veterans simultaneously felt immersed in the virtual world and that they were using VR HMDs. Median immersion ratings increased in later sessions, which indicates that immersion increased over time and technology awareness was lessened. Immersion was more variable for Oculus Rift than Gear VR. Regarding VR dose, most 20-minute sessions were rated as too short. Ratings suggest that Veterans may prefer somewhat longer sessions for Gear VR than the more immersive Oculus Rift. Minimal adverse events, including cybersickness and the weight of the Gear VR aggravating pain, were reported. Finally, more than 30% of Veterans purchased VR HMDs or requested purchasing information during the study.

### Clinical Implications

#### Aim 1

Considering the speed in which Veterans completed the hierarchy and their app intensity ratings, it is likely that the hierarchy was less incremental than designed. This would suggest that our approach to the hierarchy proved too conservative. Another possibility is that the intensity of their VR experience was shaped less by movement than their interest or engagement in the self-selected activities. The hierarchy will be modified before future use. The modification process will take a bottom-up approach to include Veteran stakeholders throughout this process of app selection, testing, and intensity rating [39]. Despite the movement heterogeneity in these Veteran-selected activities, these apps and their intensity ratings will be considered in the modification process. Having a structured app selection process for the hierarchy is important because of the goal to generalize to other VA hospitals and clinics. This is to ensure Veterans are not over- or underexposed to feared movements.

#### Aim 2

Effect sizes for kinesiophobia and pain outcomes were smaller than in previous research. It is possible that VR is more efficacious for acute than chronic pain. However, these effects may have been attenuated by the ceiling and range restriction effects in the hierarchy. Moreover, given the wide CI and the small sample, estimation of CI at a lower limit of 75% to 85% may be appropriate to complement descriptive information in VR feasibility work [68]. When considering the MCID statistics in accordance with observed effect sizes for fear of movement (FDAQ), interference with mobility, pain intensity, pain catastrophizing, and patient-specific functioning may be promising for future research. Interestingly, Veteran-selected activities hindered by pain had the largest effect sizes and proportion of MCID. As discussed previously, Veteran-selected activities produced the highest VR intensity ratings. These findings highlight the importance of considering user preferences in selecting meaningful outcomes in addition to the VR intervention itself.

#### Aim 3

Despite noted concerns with the hierarchy, this study further supports the feasibility of VR for pain management [30,69]. Specifically, the integration of VR technology itself was considered successful. Although VR was adjunct, Veterans both attended and completed sessions at high rates. Veterans typically rated VR sessions as too short, which was consistent with the success of a recent study that provided twelve 30-minute sessions using Oculus Rift [69]. It is notable that Veterans’ ratings showed marginally lower preferences for 20-minute sessions using Oculus Rift. Given that immersion was also slightly higher for Oculus Rift than Gear VR, consideration for immersion level, HMD selection [70], and sensory demand [14,32] is important when considering session length with chronic pain populations.

### Limitations

Consistent difficulties emerged when using Gear VR in this study. First, inconsistent hospital Wi-Fi hindered the importance of certain apps (eg, Guided Meditation). Second, Gear VR lacked a “kiosk mode” and apps would often time out and need to be restarted during sessions. This may have affected immersion. Third, Veterans indicated that environmental noise in the therapy gym was an issue, but only for Gear VR. This study used two Gear VR HMDs and one Oculus Rift. This approach was due to equipment availability and physical space considerations. The power supply on the gaming computer also shorted out during the first day of testing. Hence, only Gear VR was used during the first four testing days. These factors may have accelerated Veteran progress across the hierarchy. Use of a single HMD type—Oculus Rift—may be more beneficial for validation of VR hierarchies in busy medical settings.

Veterans used VR as an adjunct and were involved in on-going interdisciplinary pain management, which likely influenced Veteran improvement in treatment outcomes (see Murphy et al [36,37]). This may have also influenced Veterans’ quick progressions across the hierarchy. Inclusion of a randomized control group is necessary to estimate the true added impact of VR versus treatment as usual.

This study had additional limitations, including a small sample size, which was limited by the allotted 3-week data collection period. This likely influenced the wide CI around the effect sizes [67]. Additionally, Veterans may not have been adequately challenged by the hierarchy. Finally, because this pilot study was unfunded, commercially available VR apps were used. A more optimal approach would be to develop or tailor VR apps to capture frequently avoided movements of varying intensity levels, such as safe strategies for bending or climbing stairs.

### Conclusions

This study provided evidence that VR is feasible for chronic pain populations. In addition, this study expanded the knowledge base by demonstrating feasibility for VR as an adjunct for evidence-based chronic pain interventions in a medical setting. Implementation was more successful for VR technologies than the distraction-to-exposure hierarchy itself. Future research will focus on modification of this hierarchy to validate feared movements at varying intensity levels. This may be best accomplished using a bottom-up approach that includes Veterans in the intervention design and outcome selection processes.

## Abbreviations

CI: confidence interval
FDAQ: Fear of Daily Activities Questionnaire
HMD: head-mounted display
IQR: interquartile range
MCID: minimum clinically important difference
POQ-VA: Pain Outcomes Questionnaire-VA
PSFS: Patient-Specific Functional Scale
RCT: randomized controlled trial
VA: Veterans Affairs
VR: virtual reality

## References

[1] Dionisio JDN, Burns WG III, Gilbert R (2013). 3D Virtual worlds and the metaverse. ACM Comput Surv.

[2] Parkin S (2014). MIT Technology Review.

[3] Steuer J (1992). Defining virtual reality: dimensions determining telepresence. J Comm.

[4] Garrett B, Taverner T, Masinde W, Gromala D, Shaw C, Negraeff M (2014). A rapid evidence assessment of immersive virtual reality as an adjunct therapy in acute pain management in clinical practice. Clin J Pain.

[5] Cummings JJ, Bailenson JN (2015). How immersive is enough? A meta-analysis of the effect of immersive technology on user presence. Media Psychology.

[6] Freeman D, Reeve S, Robinson A, Ehlers A, Clark D, Spanlang B, Slater M (2017). Virtual reality in the assessment, understanding, and treatment of mental health disorders. Psychol Med.

[7] Olmos-Raya E, Ferreira-Cavalcanti J, Contero M, Castellanos MC, Giglioli IAC, Alcañiz M (2018). Mobile virtual reality as an educational platform: a pilot study on the impact of immersion and positive emotion induction in the learning process. EURASIA J Math Sci Tech Ed.

[8] Trost Z, Parsons TD (2014). Beyond distraction: virtual reality graded exposure therapy as treatment for pain-related fear and disability in chronic pain. J Appl Biobehav Res.

[9] Chan E, Foster S, Sambell R, Leong P (2018). Clinical efficacy of virtual reality for acute procedural pain management: a systematic review and meta-analysis. PLoS One.

[10] Merskey H, Bogduk N, International Association for the Study of Pain, Task Force on Taxonomy (1994). Part III: a current list with definitions and notes on usage. Classification Of Chronic Pain: Descriptions Of Chronic Pain Syndromes And Definitions Of Pain Terms.

[11] Treede R, Rief W, Barke A, Aziz Q, Bennett MI, Benoliel R, Cohen M, Evers S, Finnerup NB, First MB, Giamberardino MA, Kaasa S, Kosek E, Lavandʼhomme P, Nicholas M, Perrot S, Scholz J, Schug S, Smith BH, Svensson P, Vlaeyen JW, Wang S (2015). A classification of chronic pain for ICD-11. Pain.

[12] Grichnik KP, Ferrante FM (1991). The difference between acute and chronic pain. Mt Sinai J Med.

[13] Nijs J, Paul van Wilgen C, Van Oosterwijck J, van Ittersum M, Meeus M (2011). How to explain central sensitization to patients with 'unexplained' chronic musculoskeletal pain: practice guidelines. Man Ther.

[14] Simons LE, Elman I, Borsook D (2014). Psychological processing in chronic pain: a neural systems approach. Neurosci Biobehav Rev.

[15] Murphy J, McKellar J, Raffa S, Clark M, Kerns R, Karlin B (2014). Cognitive Behavioral Therapy for Chronic Pain Among Veterans: Therapist Manual.

[16] Lethem J, Slade P, Troup J, Bentley G (1983). Outline of a fear-avoidance model of exaggerated pain perception--I. Behav Res Ther.

[17] Vlaeyen JW, Linton SJ (2000). Fear-avoidance and its consequences in chronic musculoskeletal pain: a state of the art. Pain.

[18] Leeuw M, Goossens ME, Linton SJ, Crombez G, Boersma K, Vlaeyen JW (2007). The fear-avoidance model of musculoskeletal pain: current state of scientific evidence. J Behav Med.

[19] Linton S, Shaw W (2011). Impact of psychological factors in the experience of pain. Phys Ther.

[20] George SZ, Zeppieri G (2009). Physical therapy utilization of graded exposure for patients with low back pain. J Orthop Sports Phys Ther.

[21] Smeets RJ, Vlaeyen JW, Hidding A, Kester AD, van der Heijden GJ, van Geel AC, Knottnerus JA (2006). Active rehabilitation for chronic low back pain: cognitive-behavioral, physical, or both? First direct post-treatment results from a randomized controlled trial [ISRCTN22714229]. BMC Musculoskelet Disord.

[22] Parsons TD, Trost Z, Ma M, Jain LC, Anderson P (2014). Virtual reality-graded exposure therapy as treatment for pain-related disability in chronic pain. Virtual, Augmented Reality And Serious Games For Healthcare 1 (Intelligent Systems Reference Library).

[23] Hoffman HG, Chambers GT, Meyer WJ, Arceneaux LL, Russell WJ, Seibel EJ, Richards TL, Sharar SR, Patterson DR (2011). Virtual reality as an adjunctive non-pharmacologic analgesic for acute burn pain during medical procedures. Ann Behav Med.

[24] McCaul KD, Malott JM (1984). Distraction and coping with pain. Psychol Bull.

[25] Hoffman HG, Meyer WJ, Ramirez M, Roberts L, Seibel EJ, Atzori B, Sharar SR, Patterson DR (2014). Feasibility of articulated arm mounted Oculus Rift Virtual Reality goggles for adjunctive pain control during occupational therapy in pediatric burn patients. Cyberpsychol Behav Soc Netw.

[26] Malloy Kevin M, Milling LS (2010). The effectiveness of virtual reality distraction for pain reduction: a systematic review. Clin Psychol Rev.

[27] Wiederhold BK, Gao K, Sulea C, Wiederhold MD (2014). Virtual reality as a distraction technique in chronic pain patients. Cyberpsychol Behav Soc Netw.

[28] Jin W, Choo A, Gromala D, Shaw C, Squire P (2016). A virtual reality game for chronic pain management: a randomized, controlled clinical study. Stud Health Technol Inform.

[29] Yilmaz Yelvar GD, Çırak Y, Dalkılınç M, Parlak Demir Y, Guner Z, Boydak A (2017). Is physiotherapy integrated virtual walking effective on pain, function, and kinesiophobia in patients with non-specific low-back pain? Randomised controlled trial. Eur Spine J.

[30] Thomas JS, France CR, Applegate ME, Leitkam ST, Walkowski S (2016). Feasibility and safety of a virtual reality dodgeball intervention for chronic low back pain: a randomized clinical trial. J Pain.

[31] Garcia-Palacios A, Botella C, Hoffman H, Fabregat S (2007). Comparing acceptance and refusal rates of virtual reality exposure vs. in vivo exposure by patients with specific phobias. Cyberpsychol Behav.

[32] Parham LD, Cohn ES, Spitzer S, Koomar JA, Miller LJ, Burke JP, Brett-Green B, Mailloux Z, May-Benson TA, Roley SS, Schaaf RC, Schoen SA, Summers CA (2007). Fidelity in sensory integration intervention research. Am J Occup Ther.

[33] Garrett B, Taverner T, Gromala D, Tao G, Cordingley E, Sun C (2018). Virtual reality clinical research: promises and challenges. JMIR Serious Games.

[34] (2017). US Department of Veterans Affairs.

[35] Dahlhamer J, Lucas J, Zelaya C, Nahin R, Mackey S, DeBar L, Kerns R, Von Korff M, Porter L, Helmick C (2018). Prevalence of chronic pain and high-impact chronic pain among adults-United States, 2016. MMWR Morb Mortal Wkly Rep.

[36] Murphy JL, Clark ME, Banou E (2013). Opioid cessation and multidimensional outcomes after interdisciplinary chronic pain treatment. Clin J Pain.

[37] Murphy JL, Phillips KM, Rafie S (2016). Sex differences between Veterans participating in interdisciplinary chronic pain rehabilitation. J Rehabil Res Dev.

[38] Krebs EE, Carey TS, Weinberger M (2007). Accuracy of the pain numeric rating scale as a screening test in primary care. J Gen Intern Med.

[39] Curran GM, Bauer M, Mittman B, Pyne JM, Stetler C (2012). Effectiveness-implementation hybrid designs: combining elements of clinical effectiveness and implementation research to enhance public health impact. Med Care.

[40] Birckhead B, Khalil C, Liu X, Conovitz S, Rizzo A, Danovitch I, Bullock K, Spiegel B (2019). Recommendations for methodology of virtual reality clinical trials in health care by an international working group: iterative study. JMIR Ment Health.

[41] (2016). Guided Meditation VR.

[42] (2016). nDreams.

[43] Now VR (2016). Rest VR: Relax and Meditate.

[44] (2016). Picselica.

[45] (2017). Greener Games.

[46] (2016). Immersive Entertainment Inc.

[47] (2016). Multiverse Entertainment.

[48] (2017). Facebook.

[49] (2017). Google VR.

[50] (2017). Arrowiz.

[51] NiVision (2017). The Show Must Go On.

[52] (2016). Oculus VR.

[53] (2015). Samsung Oculus VR.

[54] Czub M, Piskorz J (2017). Body movement reduces pain intensity in virtual reality–based analgesia. Int J Hum-Comput Int.

[55] Tashjian VC, Mosadeghi S, Howard AR, Lopez M, Dupuy T, Reid M, Martinez B, Ahmed S, Dailey F, Robbins K, Rosen B, Fuller G, Danovitch I, IsHak W, Spiegel B (2017). Virtual reality for management of pain in hospitalized patients: results of a controlled trial. JMIR Ment Health.

[56] Cole J, Crowle S, Austwick G, Slater DH (2009). Exploratory findings with virtual reality for phantom limb pain; from stump motion to agency and analgesia. Disabil Rehabil.

[57] Clark ME, Gironda RJ, Young RW (2003). Development and validation of the Pain Outcomes Questionnaire-VA. J Rehabil Res Dev.

[58] George SZ, Valencia C, Zeppieri G, Robinson ME (2009). Development of a self-report measure of fearful activities for patients with low back pain: the fear of daily activities questionnaire. Phys Ther.

[59] Salaffi F, Stancati A, Silvestri CA, Ciapetti A, Grassi W (2004). Minimal clinically important changes in chronic musculoskeletal pain intensity measured on a numerical rating scale. Eur J Pain.

[60] Sullivan MJ, Bishop SR, Pivik J (1995). The Pain Catastrophizing Scale: development and validation. Psychol Assessment.

[61] Osman A, Barrios FX, Kopper BA, Hauptmann W, Jones J, O'Neill E (1997). Factor structure, reliability, and validity of the Pain Catastrophizing Scale. J Behav Med.

[62] Stratford P (1995). Assessing disability and change on individual patients: a report of a patient specific measure. Physiother Can.

[63] Abbott JH, Schmitt J (2014). Minimum important differences for the patient-specific functional scale, 4 region-specific outcome measures, and the numeric pain rating scale. J Orthop Sports Phys Ther.

[64] Kadel R, Kip K (2012). A SAS macro to compute effect size (Cohen's d) its confidence interval from raw survey data.

[65] Cohen J (1988). Statistical Power Analysis For The Behavioral Sciences (2nd Edition).

[66] Tukey JW (1977). Exploratory Data Analysis.

[67] Cohen J (1994). The earth is round (*p*<.05). Am Psychol.

[68] Lee EC, Whitehead AL, Jacques RM, Julious SA (2014). The statistical interpretation of pilot trials: should significance thresholds be reconsidered?. BMC Med Res Methodol.

[69] Garrett B, Taverner T, McDade P (2017). Virtual reality as an adjunct home therapy in chronic pain management: an exploratory study. JMIR Med Inform.

[70] Tong X, Gromala D, Gupta D, Squire P (2016). Usability comparisons of head-mounted vs. stereoscopic desktop displays in a virtual reality environment with pain patients. Stud Health Technol Inform.

